# How far can omics go in unveiling the mechanisms of floral senescence?

**DOI:** 10.1042/BST20221097

**Published:** 2023-06-30

**Authors:** Hilary J. Rogers

**Affiliations:** School of Biosciences, Cardiff University, Cardiff, U.K.

**Keywords:** floral, senescence, transcriptomics, volatilome

## Abstract

Floral senescence is of fundamental interest in understanding plant developmental regulation, it is of ecological and agricultural interest in relation to seed production, and is of key importance to the production of cut flowers. The biochemical changes occurring are well-studied and involve macromolecular breakdown and remobilisation of nutrients to developing seeds or other young organs in the plant. However, the initiation and regulation of the process and inter-organ communication remain to be fully elucidated. Although ethylene emission, which becomes autocatalytic, is a key regulator in some species, in other species it appears not to be as important. Other plant growth regulators such as cytokinins, however, seem to be important in floral senescence across both ethylene sensitive and insensitive species. Other plant growth regulators are also likely involved. Omics approaches have provided a wealth of data especially in ornamental species where genome data is lacking. Two families of transcription factors: NAC and WRKY emerge as major regulators, and omics information has been critical in understanding their functions. Future progress would greatly benefit from a single model species for understanding floral senescence; however, this is challenging due to the diversity of regulatory mechanisms. Combining omics data sets can be powerful in understanding different layers of regulation, but *in vitro* biochemical and or genetic analysis through transgenics or mutants is still needed to fully verify mechanisms and interactions between regulators.

## Introduction

**Figure 1.**
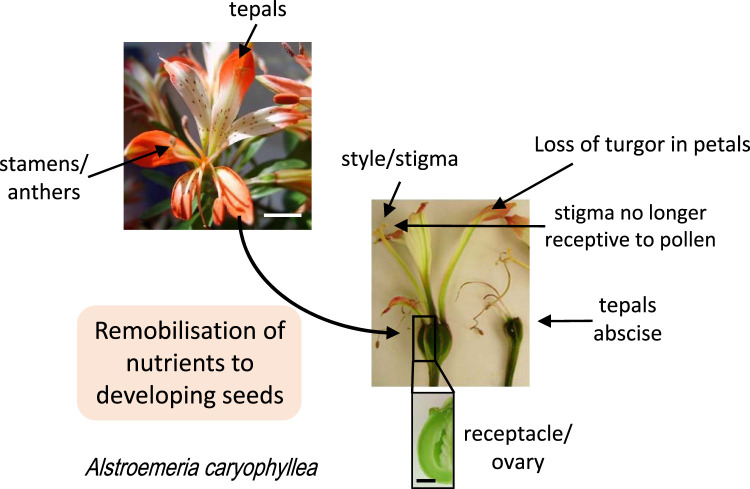
Floral senescence and remobilisation of nutrients. During floral senescence, there is a loss of turgor in the petals/sepals (in some species known as tepals) and nutrients (including amino acids, fatty acids, phosphate, sugars, minerals) are remobilised to the developing seeds within the ovary. The stigma loses receptivity to pollen, and anthers/stamens lose functionality. In many species tepals abscise. Scale bar = 10 mm. Images from [54].

Flowers are complex structures typically composed of four separate organs: sepals, leaf-like organs that protect the developing bud, petals: that act as attractants for pollinators, stamens that produce pollen and pistils that enclose the ovary in which seeds will develop. Floral senescence is a key developmental stage that allows a plant to remobilise nutrients from flowers to other organs such as the developing seeds [1] once the flower is no longer needed (Figure 1). It also reduces the risk of pathogen invasion through the stigma [2]. Maintaining a flower imposes an energetic cost on the plant, thus floral longevity is optimised to maximise seed output [3] and the stigma only remains receptive to pollen for a limited period, known as the effective pollination period [4]. Floral senescence is thus co-ordinated across the different organs and is of relevance to seed crop productivity as well as of fundamental interest in understanding plant development.

**Figure 2.**
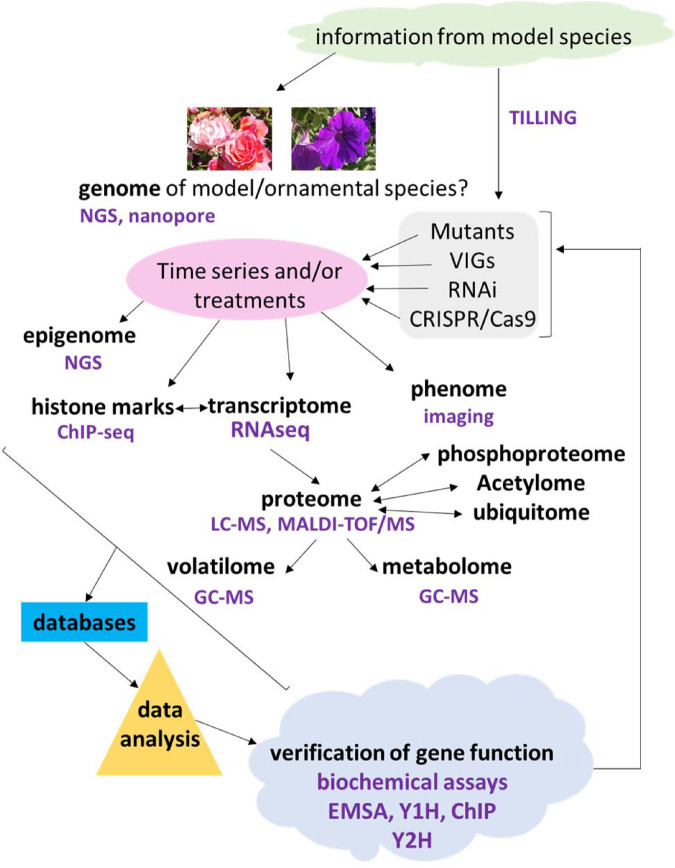
Information from a model species such as Arabidopsis can be used in ornamental species, enhanced with genome sequence data. It can be used directly via TILLING to identify mutants in homologous genes of interest. Transcriptomics, epigenomics, an analysis of the effects of histone marks and phenomics can be applied to developmental time series and/or treatments. Effects of the transcriptome can be verified in the proteome which will in turn affect the metabolome and volatilome. The effective proteome will be modulated by post-translational modifications. To exploit fully these omic datasets, comprehensive databases and powerful data analysis are required. Ultimately verification is needed via biochemical *in vitro* approaches and/or transgenics or mutants.

Model organisms such as *Arabidopsis thaliana* and rice (*Oryza sativa*), have enabled an explosion of data, and new understanding of plant developmental processes and biochemistry over the last 25 years [5]. These two species have been adopted widely as examples of the two major taxonomic groups in plants, dicots and monocots, respectively. This has been at least in part enabled by the development of omics approaches, in which the chief players have been genomics, transcriptomics, proteomics and metabolomics [6]. However, recent omics additions of volatilomics, epigenomics, methylomics and lipidomics, amongst others, are expanding the data types and adding considerably to the original omics frameworks [7] (Figure 2). These add important tools which can be combined to create better models of gene function and biochemical processes, especially through the increasing number of databases and rapid progress in data analysis. Moreover, combination of omics tools with classical genetics, creation of transgenic plants, and high throughput phenotyping (phenomics) has enables the link between phenotype and genotype to be rapid and informative. This allows forward (mutant screening) and reverse (targeting specific genes using transgenics) genetics to pinpoint specific biochemical pathways and regulatory modules in complex organs such as flowers. However, neither Arabidopsis nor rice provide an ideal model for floral senescence.

## Do we need models for floral senescence?

One of the key difficulties facing the floral senescence community has been the identification of a suitable model. Floral senescence across angiosperms is diverse and falls into two major biochemical divisions which are partially but not completely associated with taxonomy [8]. In one major group of flowers, ethylene is a major regulator of floral senescence: this includes major ornamental species such as carnations and many roses. These flowers are characterised by a peak of ethylene biosynthesis, often activated by pollination and fertilisation, which becomes autocatalytic [9]. This activates key enzymes in petal cells responsible for the breakdown of the major cellular macromolecules and ordered destruction of organelles, through established signalling pathways [9,13]. In fertilised flowers, the sugars, amino acids and other nutrients including phosphates and other key metabolites are then remobilised to the developing ovary and seeds. When senescence is activated without fertilisation, remobilisation is to other parts of the plant such as younger flowers or shoots [1,10]. In another group of species, including lilies and composite flowers such as chrysanthemums and dahlias, ethylene seems to play a much more minor role in floral senescence, although downstream enzyme activation and cellular disassembly is very similar [11,12]. In contrast, another phytohormone class, cytokinins, seem to delay floral senescence both in ethylene sensitive and ethylene-insensitive species [13]. In ethylene-sensitive flowers, cytokinins seem to act by inhibiting ethylene synthesis formation of reactive oxygen species [14], while in ethylene-insensitive petal senescence, cytokinins may act by increasing the levels of antioxidants [15]. Another major divide in floral senescence phenotype is between petals that lose turgor and wilt while still attached to the flower such as daffodils and petunias, and those in which the petal is abscised from the flower still relatively turgid such as magnolias and most tulips [16].

Arabidopsis belongs to the ethylene-regulated group whose petals abscise turgid [17] and has been useful in understanding many aspects of floral biology through the multiple omics and mutant resources available. Early transcriptomic studies enabled useful comparisons between petal senescence and senescence in other, better-studied organs [18]. More recently important transcription factors regulating ethylene-dependent senescence have been identified through mutant analysis, based on the Arabidopsis genome sequence, and complex regulatory networks have been identified through reverse genetics. For example, *FOREVER YOUNG FLOWER* (*FYF*) suppresses downstream ethylene signalling regulators, but FYF-like genes, have evolved to suppress or promote flower senescence and regulate abscission through the formation of heterotetrameric abscission complexes with other MADS box transcription factors [19]. However, Arabidopsis has small self-fertile flowers in which the progression of senescence is not very easily staged. In addition, it is not a commercial crop, either as an ornamental or for food use. To make better use of forward genetics screens in more tractable model species methods such as targeting-induced local lesions in genomes (TILLING) would be helpful in which populations of mutants can be screened for single nucleotide polymorphisms (SNPs) in key genes identified in the models [20].

## Floral omics in ornamental species

Substantial progress has been made in the field of floral senescence through the study of a few important ornamental crops, most importantly carnations, petunias, Japanese morning glory (*Ipomoea nil*) and roses. The publication of genome sequences for these species relatively recently [21–24] will enhance the use of these species as models for floral senescence [25]. Moreover, these species are all transformable [26] which facilitates the verification of gene functions. Transient transformation is also a useful tool in petunia using VIGS (virus-induced gene silencing; [27]). However, the scientific communities studying these species are relatively small and these species lack the rapid generation time, wealth of mutant resources and rapid transformation systems that make Arabidopsis such a good model. In addition, there is a lack of substantial progress in species with ethylene-insensitive floral senescence.

Major advances in our understanding of ethylene-sensitive floral senescence from the study of petunias has been in the interactions between ethylene and cytokinin through the use of transgenics in which cytokinin production was switched on by a senescence-induced promoter. Elevated cytokinin reduced both ethylene sensitivity and the accumulation of abscisic acid (ABA), another phytohormone that accelerates floral senescence, by promoting the production of endogenous ethylene in the flowers [28]. Revisiting the effects of cytokinin using transcriptomics revealed how rapidly changes in global gene expression occur (within 3 h) in response to exogenous cytokinin treatment, and also showed how cytokinin regulates both ABA synthesis and degradation [29]. Transcriptomics can also be used to identify how regulatory genes that have been better characterised in models, such as Arabidopsis leaf senescence, apply to the floral senescence of ornamental species. For example, an analysis of WRKY TFs in petunia identified specific members of the gene family likely associated with petunia corolla senescence [30]. Downstream from transcriptomes, proteomes are also clearly important in understanding gene function. Indeed beyond the proteome, the post-translational modification of proteins such as acetylation can be studied at the omic level. This approach was recently applied to understanding the effect of perturbing acetylation in petunia corollas through the silencing of ATP-citrate lyase genes [31]. This revealed that these genes are important metabolic regulators maintaining metabolic homeostasis in flowers as well as other plant organs.

In roses, recent advances in understanding phytohormone regulation and key transcription factors has rested on the identification of genes through homology to Arabidopsis, coupled with transgenics [13]. Large datasets of rose ESTs combined with gene silencing approaches enabled the identification of key transcription factors such as *RhHB1* which regulate both ethylene and ABA-induced senescence [32]. In addition, in varieties with pigmented petals such as *Rosa hybrida* ‘Samantha’, petals change colour as they senescence and this was exploited by using petal discs floated on different hormone solutions, an approach which would not have been possible in Arabidopsis.

In addition to the transfer of understanding from models such as Arabidopsis, omics also enables the transfer of understanding between different ornamental species. For example, two transcription factors, *DcWRKY75* and *DcHB30* were recently identified in carnation, which together regulate ethylene-induced petal senescence [33,34]. Both were identified through transcriptome analysis and selected on the basis of expression pattern clustering through a senescence time-course, emphasising the power of detailed time courses for assessing gene function. WRKY TFs are already known to be important regulators of Arabidopsis leaf senescence [35]. However, HB transcription factors had already been identified in both rose and petunia as important in the regulation of floral senescence [32,36] and hence this information across different ornamental species was used in the selection of *DcHB30* for further study.

A major strength of omics approaches is also their versatility and use in species for which there is much more limited knowledge of genetics. Lilies have some of the largest genomes of any organism (up to 168 pg; [37]), which has delayed genome sequencing while still allowing transcriptomic approaches. Even in these monocotyledonous species which are taxonomically quite distant from Arabidopsis, it has been possible to use gene sequence knowledge and signalling pathways deciphered in Arabidopsis through omics approaches to inform floral senescence. For example, the role of auxin-responsive ARF transcription factors in regulating auxin inhibition of lily petal abscission was inferred through obtaining target sequences from a transcriptome and comparison of gene expression in contrasting lily species where petals abscise turgid or remain attached to the flower [38].

Transcriptomics is also helping to understand senescence in complex floral architectures such as those of composite chrysanthemums and dahlias [39,40]. These flowers are made up of hundreds of individual florets attached to a central receptacle. Floret age increases from the centre of the flower head to the edge, thus floral senescence is a careful coordination of senescence in a gradient across the flower but also temporally as the whole flower head ages. Transcriptomics has shown that 35 different transcription factor families are involved in the regulation of dahlia floret senescence [39] with most changes in expression seen between inner and outer florets rather than during flower head aging although some are activated in both. However, one of the complications in interpreting these data is in ascribing functions to specific genes since the genome of ornamental dahlias is octoploid, hence there are potentially many different homeologues of each Arabidopsis gene [39].

## Combining omics

Whereas many earlier studies on floral senescence were mainly based on transcriptomics, more recently combinations of omics are being applied revealing unexpected complexity (Figure 2). For example, combining proteome and transcriptome in chrysanthemum showed diverging results [40]. This is thought to be due to the important role of ubiquitination in floral senescence confirmed also in petunia [41] and rose [42], that changes proteome representation.

Advances in the sensitivity volatile organic compound (VOC) detection have enabled a flourishing of floral volatilomics [43]. Floral scent is a key component of the pollination syndrome that tailors floral attraction to pollinators. This includes circadian regulation of floral scent production and targeted reduction in VOC biosynthesis as flowers age and pollination occurs [44]. For example in the flowers of *Eurya japonica* which is a subdioecious species with individuals producing male, female or hermaphrodite flowers, the emission of VOCs decreases in male flowers after pollination as the flowers senescence but does not in female or hermaphrodite flowers. This is presumed to be to reduce the energetic costs in male plants [45]. Combined with transcriptome and genome information, volatilomics is enabling major advances in understanding the transcriptional regulation and biosynthetic pathways regulating floral scent production [43,44]. For example, transcriptomics in species producing scented flowers is exploring the wide variation in the >2000 terpene synthases (TPS) identified to date, responsible for the 1000s of mono and sesqui terpenes in floral scent bouquets [44]. Omics tools are also enabling the identification of the transcription factors from e.g. MYB, WRKY and bHLH families regulating the expression of the TPS genes. It is important to note though that the precise activity of each TPS still needs to be verified through transgenic analysis or through *in vitro* biochemical assays [46].

In tuberose (*Agave amica*) multivariate analysis of transcriptomes, metabolomes and volatilomes showed a synchronisation between tABC transporter gene expression and emission of terpene VOCs [47]. This study also linked changes in phenylpropanoid to flavonoid metabolism to specific enzyme isoforms and transcription factors. As flowers entered senescence and VOC emission declined there was an increase in VOC glycosylation perhaps linked to their storage and export from the flower.

Another powerful combination of omics can be the comparison of transcriptome, proteome and post-translational modification of proteins such as the acetylome and metabolome. In petunia, these three omics approaches were used to confirm that silencing of ACL genes, which accelerates floral senescence resulted in changes to the transcription of secondary metabolite biosynthesis genes, which was confirmed through changes in the proteome and metabolome [31].

Finally and importantly, mutational screening remains the key tool in use for ornamental breeding [20]. This is of particular importance as so many important ornamental species remain refractory to genetic transformation. A range of methods are available for random mutagenesis, including chemical and radiation-based methods. Combined with TILLING populations, this classical approach could be significantly enhanced, linking it to the wealth of information from omics approaches. However, it will require substantial effort in checking that the gene function identified through model species is indeed equivalent in the ornamental species.

## Floral senescence involves inter-organ coordination

The majority of research on floral senescence has focussed on petals as these are of particular interest to the ornamental industry. However, a key feature of floral senescence is the coordination of events across the different organs. Transcriptomics have helped here in understanding how genes identified as important in the regulation of leaf and petal senescence may also play a role in the senescence of other floral organs.

For example, the NAC family transcription factor *ANAC092* previously identified as a leaf senescence regulator was identified also through transcriptomic analysis of senescent stigmas and found to act together with *ANAC074* to regulated cell death genes that terminate the life of Arabidopsis stigma [48]. Understanding the control of stigma receptivity may be important in optimising fruit set under environmental stress. Transcriptomic comparisons of senescence in different floral organs also show that some of the pathways activated are organ specific. A comparison of the transcriptomes of senescent petals, style + stigma + stamens and ovaries in short-lived *Hibiscus rosa-sinensis* flowers revealed many more changes in expression in petals, style + stigma + stamens compared with ovaries [49]. For example, expression of the blue light receptor *PHOTOTROPIN 1* (*PHOT1*) was up-regulated in the petals and style + stigma + stamen suggesting light/circadian clock regulation of senescence in this flower. Interestingly circadian control seems to also be important in longer-lived flowers such as rose [50]. Transcriptomic analysis identified a B-box transcription factor, *RhBBX28* that regulates floral senescence and night time mitochondrial ROS homeostasis. In turn, this gene is regulated by interaction with *PHYTOCHROME-INTERACTING FACTOR8* (*RhPIF8*).

## Limitations and future potential of the omics approaches

The combination of omics approaches is extremely powerful in discovering new pathways that may be important in regulating complex processes such as floral senescence. One key to full understanding is in large datasets, for example with highly detailed time courses. These have been important in leaf senescence in revealing the exact sequences of pathways activated and thus understanding causal relationships [51]. Another important factor is the application of multivariate statistical approaches and machine learning, combining new data and meta analyses of published datasets to unravel gene networks and develop systems biology models, e.g. [52,53]. In floral senescence, the lack of a perfect model and the cross-species differences in senescence regulation has hindered some of these approaches as it has been difficult to build these very large datasets and resources.

The biggest limitation to exploiting omics data ultimately is in verifying metabolite identities and gene functions. The most frequent next step from transcriptome is to infer gene function by homology to database sequences. However, this relies on genetic or biochemical evidence from different species and often different plan organs so needs to be treated with caution. Testing through mutants and transgenics is much easier in models such as Arabidopsis where transformation is routine and large sets of mutants and other resources are widely available. In transformable species such as rose and petunia CRISPR–Cas9 will enable much more rapid verification by generating targeted mutants which previously was not achievable in plants, and already this approach has been applied to verify floral senescence regulators such as the NAC transcription factor *EPHEMERAL 1* in Japanese morning glory (*Ipomoea nil*.; [54]). However, for verification of some protein and gene functions *in vitro* assays may be more useful. These have the advantage that they can be applied also to non-transformable species, but are often quite laborious. For metabolites, there is still a serious lack of comprehensive and easily searchable databases to make best use of the data although progress is being made [55].

Another limitation in much of the omic work on flower senescence is the lack of data for individual cell types or even regions of individual floral organs. Even petals, which are relatively simple, develop differently in their edge and midrib and tip and base as well as showing differences between epidermal and inner structures. Laser microdissection and fluorescence-activated cell sorting (FACS) approaches, together with single-cell transcriptomics [56] will enable this dimension to be better explored.

Finally, it is important in a field that focusses on ornamental flowers that the information gained can be applied to the commercial sector through breeding, treatments to improve longevity or gene editing. Interaction with the industry will ensure that the omics data gained is not only of fundamental interest but also of practical relevance.

## Perspectives

- Flower senescence is of relevance to ornamental flower production, reproductive ecology but also to our understanding of a fundamental plant developmental process.
- Omics has opened the way to study floral senescence in a myriad of different ornamental species but without a common model progress is slow. The diversity in floral senescence hampers the identification of a suitable model system.
- Key steps are the integration of omics datasets within species and databases that can facilitate cross-species information. Another major step will be a spatial understanding of floral organ senescence across organs and within individual cells.

## Open Access

Open access for this article was enabled by the participation of Cardiff University in an all-inclusive *Read & Publish* agreement with Portland Press and the Biochemical Society under a transformative agreement with JISC.

## Abbreviations

ABA: abscisic acid
TILLING: targeting-induced local lesions in genomes
TPS: terpene synthases
